# Hydrogen Sulfide Mitigates Chilling Injury of Postharvest Banana Fruits by Regulating γ-Aminobutyric Acid Shunt Pathway and Ascorbate–Glutathione Cycle

**DOI:** 10.3389/fpls.2022.941246

**Published:** 2022-07-05

**Authors:** Sajid Ali, Aamir Nawaz, Safina Naz, Shaghef Ejaz, Mehdi Maqbool, Manzer H. Siddiqui, Hazem M. Kalaji, Jacek Wróbel, Arkadiusz Telesiński, Alicja Auriga

**Affiliations:** ^1^Department of Horticulture, Faculty of Agricultural Sciences and Technology, Bahauddin Zakariya University, Multan, Pakistan; ^2^Department of Horticulture, University of Poonch Rawalakot, Rawalakot, Pakistan; ^3^Department of Botany and Microbiology, College of Science, King Saud University, Riyadh, Saudi Arabia; ^4^Institute of Technology and Life Sciences – National Research Institute, Falenty, Poland; ^5^Department of Plant Physiology, Institute of Biology, Warsaw University of Life Sciences – SGGW, Warsaw, Poland; ^6^Department of Bioengineering, West Pomeranian University of Technology, Szczecin, Poland; ^7^Department of Animal Anatomy and Zoology, Faculty of Biotechnoology and Animal Husbandary, West Pomeranin University of Technology, Szczecin, Poland

**Keywords:** antioxidant activities, chilling injury, cold storage, lipid peroxidation, proline accumulation

## Abstract

This study aimed to determine the effect of hydrogen sulfide on chilling injury (CI) of banana (*Musa* spp.) during cold storage (7°C). It was observed that hydrogen sulfide application (2 mmol L^–1^) markedly reduced the CI index and showed significantly higher chlorophyll contents, along with suppressed chlorophyll peroxidase and chlorophyllase enzyme activity. The treated banana fruits exhibited substantially higher peel lightness (L*), along with significantly a lower browning degree and soluble quinone content. The treated bananas had substantially a higher endogenous hydrogen sulfide content and higher activity of its biosynthesis-associated enzymes such as D-cysteine desulfhydrase (DCD) and L-cysteine desulfhydrase (LCD), along with significantly lower ion leakage, lipid peroxidation, hydrogen peroxide, and superoxide anion concentrations. Hydrogen sulfide-treated banana fruits showed an increased proline content and proline metabolism-associated enzymes including ornithine aminotransferase (OAT), Δ^1^-pyrroline-5-carboxylate synthetase (P5CS), and proline dehydrogenase (PDH). In the same way, hydrogen sulfide-fumigated banana fruits accumulated higher endogenous γ-aminobutyric acid (GABA) due to enhanced activity of glutamate decarboxylase (GAD) and GABA transaminase (GABA-T) enzymes. The hydrogen sulfide-treated fruits exhibited higher total phenolics owing to lower polyphenol oxidase (PPO) and peroxidase (POD) activity and stimulated phenylalanine ammonia lyase (PAL). The treated banana exhibited higher ascorbate peroxidase (APX), catalase (CAT), glutathione reductase (GR), dehydroascorbate reductase (DHAR), monodehydroascorbate reductase (MDHAR), and superoxide dismutase (SOD) activity, along with higher glutathione (GSH) and ascorbic acid (AsA) concentrations and a significantly lower dehydroascorbic acid (DHA) content. In conclusion, hydrogen sulfide treatment could be utilized for CI alleviation of banana fruits during cold storage.

## Introduction

Cold storage has been widely used to increase the shelf life of horticultural commodities owing to its potential in suppressing metabolic processes (Zhang et al., 2021). However, chilling injury (CI) occurs in certain susceptible horticultural commodities of sub-tropical and tropical regions including bananas. Banana fruits are vulnerable to CI if kept under cold storage below 12°C (Chen et al., 2008). Excessive peel pitting, browning (surface discoloration), chlorophyll degradation, and failure of fruits to ripen are some prominent symptoms of CI in banana (Jiang et al., 2004). The activities of antioxidant enzymes, membrane integrity, lipid peroxidation, antioxidant capacity, and reactive oxygen species (ROS) production are the main factors that determine the storage potential and quality attributes in bananas under chilling stress. CI causes excessive ROS production in bananas, which lowers their storage and shelf life potential due to oxidative stress. In addition, the decline in consumer acceptance and marketing value takes place as a result of quality deterioration due to CI in banana fruits (Promyou et al., 2008). So, some appropriate eco-friendly approaches are needed for banana fruits to lower CI symptoms during cold storage without compromising their eating quality.

Many chemicals have been used to lower CI in banana fruits during postharvest chilling stress. Salicylic acid treatment was effective in suppressing polyphenol oxidase (PPO) enzyme activity and electrolyte leakage, which maintained better fruit quality of banana fruits with fewer CI symptoms under chilling stress conditions (Khademi et al., 2019). Fibroin treatment of banana fruits markedly reduced CI and inhibited the increase in membrane leakage of peel tissues (Liu et al., 2019). Similarly, γ-aminobutyric acid (GABA) application enhanced phenolic content biosynthesis and proline accumulation in the treated banana fruits with suppressed electrolyte leakage, malondialdehyde (MDA) content, and CI index, which thereby improved their chilling tolerance (Wang et al., 2014). Furthermore, the application of GABA efficiently reduced the overproduction of ROS, along with noticeably higher APX, CAT, and superoxide dismutase (SOD) enzyme activity. In the same way, exogenous application of progesterone (Hao et al., 2019), melatonin (Wang et al., 2021), glycine betaine (Chen et al., 2021), astragalus polysaccharides (Tian et al., 2022), phytosulfokine α (Wang et al., 2022), and ethylene (Zhou et al., 2022) have also been used for CI amelioration in banana fruits. However, some potential anti-chilling agents are needed for the mitigation of CI in low-temperature stored bananas.

Hydrogen sulfide like carbon monoxide (CO) and nitric oxide (NO) is an important gaseous transmitter. It is considered an imperative signaling molecule that regulates numerous physiological functions in plants. It plays a critical role in coping with various stresses and helps plants to withstand certain unfavorable conditions (Huang et al., 2021). Its application has also been reported in the postharvest storage of vegetables and fruits (Ali et al., 2019; Huang et al., 2022). The exogenous application of hydrogen sulfide alleviated the oxidative stress in fresh-cut lotus root slices by inhibiting the browning incidence (Sun et al., 2015). The level of the cellular energy status of the commodity during postharvest storage directly correlates with storage life potential under chilling stress. The exogenous hydrogen sulfide application was effective to maintain a higher ATP level, which subsequently lowered the respiration rate. This reduction in the respiration rate ultimately delayed the yellowing of spinach leaves during cold storage (Hu H. et al., 2015). Similarly, hydrogen sulfide maintained better membrane integrity, which therefore suppressed electrolyte leakage during chilling stress and reduced CI symptoms with suppressed ROS production in harvested eggplant fruits (Barzegar et al., 2021). The application of hydrogen sulfide has been reported on bananas in relation to energy metabolism under chilling stress (Li et al., 2016). It was observed that hydrogen sulfide treatment increased the activity of Ca^2+^-ATPase, cytochrome C oxidase, and H^+^-ATPase, which resulted in the delayed loss of cellular energy and markedly enhanced the chilling tolerance of banana fruits (Li et al., 2016). Nevertheless, the detailed work regarding the impact of hydrogen sulfide in the mitigation of banana fruit CI in relation to chlorophyll degradation, hydrogen sulfide biosynthesis-related enzymes, GABA shunt pathway, and proline metabolism and ascorbate glutathione cycle-associated enzymes has not been reported yet. So, our objective was to explore the influence of hydrogen sulfide treatment on CI, lipid peroxidation, oxidative stress, chlorophyll degradation, hydrogen sulfide biosynthesis-related enzyme activity, GABA shunt pathway, proline metabolism, and ascorbate glutathione cycle of banana fruits under postharvest chilling stress.

## Materials and Methods

### Experimental Fruit

Banana fruits (*Musa* spp., cv “Basrai,” AAA group) were obtained from the commercial plantation in Mirpur Khas, Sindh, Pakistan. The fruits were hand-harvested at the physiologically mature green stage and shifted to the laboratory [at 15°C with 85 ± 2% relative humidity (RH) within 12 h of the fruit harvest] for the research trial. The defected or bruised fruits were excluded, and only healthy with acceptable cosmetic quality were selected and used for the research purpose. The fruits were fumigated with 2 mmol L^–1^ (optimized in a preliminary work where 0.25, 0.5, 1, and 2 mmol L^–1^ was used) hydrogen sulfide [released from NaHS [a hydrogen sulfide donor (CAS Number: 207683-19-0, Sigma Aldrich, St Louis, MO, United States)] for 24 h at 20 ± 1°C with 85 ± 2% RH in a 20-L sealed jar. The control banana fruits were also kept in the sealed jar for 24 h as specified for the hydrogen sulfide treatment without NaHS. Both banana groups were then removed from the jars and immediately stored at 7°C with 90 ± 2% relative humidity for 10 days period. The bananas were then packed in cardboard boxes with a size of W = 25 cm, L = 40 cm, and H = 16 cm. The banana fruits were sampled on days 0 (before hydrogen sulfide fumigation), 2, 4, 6, 8, and 10 for various parameters or stored at −80°C after treating with liquid nitrogen for different measurements. The experiment was conducted as a completely randomized design under the factorial scheme. There were 3 replications in each treatment, and 20 banana fruits were present in every replication. The following attributes were studied during the current research trial.

### Banana Chilling Injury Index and Chlorophyll Contents in Peel Tissues

It was determined on a visual observation basis as described by Chen et al. (2021) with minor modifications. The CI was observed in the form of pitting and browning on the banana peel surface. The CI scale was as 1 = no CI symptoms, 2 = 1–25% browning, 3 = 26–50% browning, 4 = 51–75% browning, and 5 = 76–100% browning.

Chlorophyll (Chl) contents (Chl a, Chl b, and total Chl) were estimated as described previously (Lichtenthaler and Wellburn, 1983). The concentrations of Chl a, Chl b, and total Chl contents were expressed as mg kg^–1^ fresh weight (FW).

### Chlorophyll Peroxidase and Chlorophyllase Activity Determination in Peel Tissues

The activity of chlorophyllase and chlorophyll peroxidase enzymes was assayed using earlier protocols (Yamauchi et al., 1997; Pongprasert et al., 2011). A measure of 0.5 g banana peel was homogenized in phosphate buffer (100 mmol L^–1^, 7.0 pH) containing polyvinylpyrrolidone (PVP). The resultant homogenates were filtered and centrifuged for 15 min at 16,000 × *g* at 4°C. For chlorophyllase, the reaction assay consisted of 0.1 mmol L^–1^ phosphate buffer (7.5 pH), 0.5 mL enzyme extract, and 0.2 mL of 500 μg mL^–1^ acetone-chl a solution. In case of chlorophyll peroxidase, the assay mixture consisted of 1% Triton-X (0.1 mL), 0.5 mL enzymes extracts, 1.5 mL phosphate buffer (0.1 mmol L^–1^, 5.0 pH), and 0.3% of 0.1 mL H_2_O_2_, and activity was derived as U kg^–1^ protein after noting optical density at 667 nm.

### Peel Lightness, Browning Degree, and Soluble Quinone Content in Peel Tissues

Peel lightness [(L*) peel surface color] was determined with a CR-400 chroma meter (Minolta, Osaka, Japan). The color was assessed three times from equidistant points from a single fruit. A total of 20 bananas were used for L* measurement in each replication, with 60 bananas per treatment.

The banana fruit peel browning degree was determined following the protocols of Zhang et al. (2005) with a slight modification. In brief, banana peel samples (1 g) were macerated in ethanol (60% concentrated and 5 mL) and 0.1 mol L^–1^ concentrated phosphate buffer with pH 6.8, which contained 2% PVP. The homogenates were centrifuged for 15 min at 15,000 × *g* at 4°C, and supernatant absorbance was noted at 420 nm. Finally, the browning degree was expressed on an FW basis as OD_420_ g^–1^.

We determined the soluble quinone content in banana peel tissues using the protocol of Banerjee et al. (2015). Briefly, 5 g banana peel samples were homogenized in methanol (10 mL) and centrifuged (Z326-K, Hermle, Germany) for 20 min at 12,000 × *g* under 4°C conditions. The optical density of the resultant supernatant was measured at 437 nm on a UV–Vis spectrophotometer (UV-1800, Shimadzu, Japan), and the soluble quinone content was expressed as OD_437_ g^–1^ FW.

### Hydrogen Sulfide Content and Its Metabolism-Associated Enzyme Activity in Peel Tissues

Endogenous hydrogen sulfide was determined by a methylene blue assay (Zhu et al., 2018). A measure of 1 g banana peel tissue samples were extracted with PBS (6.8 pH, 50 mmol L^–1^, 2 mL), which contained 0.1 mmol L^–1^ Na_2_-EDTA and 200 mmol L^–1^ L-ascorbic acid. The subsequent homogenate was centrifuged for 10 min at 20,000 × *g* at 4°C. Thereafter, 1 mL supernatant was incorporated into a test tube which contained 10 mmol L^–1^ L-cysteine, 0.1% zinc acetate (200 μL), 2 mL PBS solution (7.4 pH, 100 mmol L^–1^), and 200 μL phosphopyridoxal (2 mmol L^–1^). The reactions in the assay mixtures were terminated after 15 min by incorporation of 150 μL dimethyl phenylenediamine, which was dissolved in H_2_SO_4_ (3.5 mmol L^–1^). Afterward, ferric ammonium sulfate was added into 3.5 mmol L^–1^ H_2_SO_4_ and left at 25°C for 15 min. Finally, the hydrogen sulfide content was denoted as μmol kg^–1^ min^–1^ after noting the optical density at 765 nm.

The activity of DCD and LCD enzyme was analyzed following detailed protocols of Riemenschneider et al. (2005). The liquid nitrogen-treated banana peel samples (1 g) were extracted at 8.0 pH and 20 mmol L^–1^ Tris–HCl buffer. The banana peel homogenates were centrifuged for 20 min at 12,000 × *g*. For the assay of DCD and LCD enzymes, the supernatant (1 mL) was reacted with 1 mL of 100 mmol L^–1^ concentrated Tris–HCl buffer (pH 8.0 for DCD and pH 9.0 for LCD), 0.8 mmol L^–1^ D-cysteine (for DCD) and L-cysteine (for LCD), and 2.5 mL of dithiothreitol. The absorbance was noted at 670 nm after the termination of the reaction by adding 30 mmol L^–1^ FeCl_3_ which was dissolved in 7.2 molar hydrochloric acid solution. The activities of DCD and LCD enzymes were expressed as μmol min kg^–1^.

### Ion Leakage and Malondialdehyde Content in Peel Tissues

Ion leakage of banana peel tissues was analyzed following the procedure of Chen et al. (2008) and expressed in terms of percent. MDA in banana peel samples was assessed with assay of Shi et al. (2018). Peel tissue homogenates were centrifuged at 10,000 × *g* for 20 min after homogenization in trichloroacetic acid (TCA). After this, thiobarbituric acid and the homogenized mixtures of the samples were boiled at 100°C for 20 min. The supernatant was collected, and MDA was denoted as nmol kg^–1^ FW after observing the optical density at 450, 532, and 600 nm on a UV–Vis spectrophotometer (UV-1800, Shimadzu, Japan).

### Lipoxygenase Enzyme Assay in Peel Tissues

The activity of lipoxygenases (LOX) was measured with detailed protocols of Liu et al. (2011). The peel samples were macerated in phosphate buffer and centrifuged at 10,000 × *g*. The obtained supernatant was reacted with sodium linoleic acid in an assay mixture of phosphate buffer. The absorbance of the assay mixture was observed at 234 nm, and LOX activity was reported as U kg^–1^ protein.

### Superoxide Anion and Hydrogen Peroxide in Peel Tissues

The assay of Yang et al. (2011) was utilized for the determination of the superoxide anion content. A measure of 1 g peel tissue samples were macerated in phosphate buffer and centrifuged at 10,000 × *g* for 15 min, and it was expressed as nmol min^–1^ kg^–1^ FW after absorbance measurement at 530 nm. Velikova et al. (2000)’s method was used for hydrogen peroxide assay. The peel samples were ground in TCA. The samples were then mixed with phosphate buffer and potassium iodide. Finally, hydrogen peroxide was derived as μmol kg^–1^ after observing optical density at 340 nm.

### γ-Aminobutyric Acid Shunt Pathway in Peel Tissues

#### γ-Aminobutyric Acid Content

The GABA content in the peel of bananas was determined by using Hu X. et al. (2015)’s assay. Tissues (1 g) were ground in 3 mL lanthanum chloride (0.05 mmol L^–1^) and were subjected to centrifugation at 13,000 × *g* for 5 min. The supernatant (400 μL) was reacted with 200 μL phenol (6%), NaOCl (5%) and phosphate buffer (0.05 mol L^–1^, 10.0 pH). The absorbance was measured at 645 nm, and it was expressed as mg kg^–1^ FW.

#### Glutamate Decarboxylase and γ-Aminobutyric Acid Transaminase Activity in Peel Tissues

The glutamate decarboxylase (GAD) and GABA-T activity was assayed following the detailed procedure of Deewatthanawong et al. (2010). Sample tissues (1 g) were ground in Tris–HCl buffer, and the supernatant was used for the activity of GAD and GABA-T enzymes. The activity of each enzyme was expressed as U mg^–1^ protein after noting optical density at 340 nm. The measurements were performed in triplicates, and all steps were carried out at 4°C.

### Proline Metabolism in Peel Tissues

#### Proline Content

Banana peel proline was estimated using earlier protocols (Shang et al., 2011). A measure of 5 mL sulfosalicylic acid was used for sample (1 g) maceration and later subjected to centrifugation at 12,000 × *g*; 3 mL ninhydrin and 2 mL acetic acid were mixed and boiled for 60 min at 100°C. The resultant homogenate was mixed with toluene. Finally, it was expressed as mg kg^–1^ FW after optical density measurement at 520 nm.

#### Δ^1^-Pyrroline-5-Carboxylate Synthetase Enzyme Activity

In the case of P5CS assay, banana peel tissues were ground in Tris–HCl buffer (7.5 pH and 50 mmol L^–1^). The activity was derived as U kg^–1^ protein after measurement of optical density at 340 nm (López-Carrión et al., 2008).

#### Ornithine Aminotransferase Enzyme Activity

The ornithine aminotransferase (OAT) activity was analyzed following the procedure of Lutts et al. (1999). The peel tissues were homogenized in Tris–HCl buffer (50 mmol L^–1^, 7.5 pH), and absorbance was noted at 510 nm. The activity of OAT was denoted as U kg^–1^ protein.

#### Proline Dehydrogenase Enzyme Activity

The proline dehydrogenase (PDH) assay was carried out according to the procedure of López-Carrión et al. (2008). The sample tissues were ground in phosphate buffer. The supernatant was collected and added to a carbonate–bicarbonate buffer containing proline. The activity of PDH was expressed as U kg^–1^ protein after noting optical density at 340 nm.

### Phenol Metabolism in Peel Tissues

#### Total Phenolic Contents

The total phenolic contents were determined with Folin–Ciocalteu (10%) reagent (Chen et al., 2021). The peel samples (2 g) were ground in a 5 mL methanolic (95%) extraction mixture, and the supernatant was collected after centrifugation at 12,000 × *g*. The assay mixture was reacted with sodium carbonate (20%) and left at room temperature after vortexing for 60 min. Finally, total phenolic contents were derived in terms of gallic acid equivalents as mg kg^–1^ FW after noting the absorbance at 765 nm on a UV–Vis spectrophotometer (UV-1800, Shimadzu, Japan).

#### Peroxidase, Polyphenol Oxidase, and Phenylalanine Ammonia Lyase Enzyme Activity

The peroxidase (POD) activity was assayed at 470 nm with a detailed protocol of Ali et al. (2016). The activity of PPO and phenylalanine ammonia lyase (PAL) was assayed as outlined by Nguyen et al. (2003). PPO activity was derived as U kg^–1^ protein after noting the optical density at 420 nm. PAL activity was derived as U kg^–1^ protein after optical density reading at 290 nm. The activity of all the aforementioned enzymes was assayed in triplicates, and all steps were carried out at 4°C conditions.

### Assay of Glutathione, Ascorbic Acid, and Dehydroascorbic Acid Content in Peel Tissues

The peel (5 g) tissues were ground in 5% ice-cold TCA solution which contained EDTA-Na_2_ (Nie et al., 2020). The supernatant (400 μL), 1 mL phosphate buffer (8.0 pH and 0.1 molar), and 4 mmol L^–1^ 600 μL of 5,5′-dithio-bis-(2-nitrobenzoic acid) were reacted. After thorough mixing of all reactants, the absorbance was noted at 412 nm, and the glutathione (GSH) content was expressed as mg kg^–1^ FW. The ascorbic acid (AsA) in banana peel was assayed following a detailed method described previously (Li et al., 2021). The sample tissues (1.5 g) were very finely macerated in 5% TCA mixture and centrifuged for 15 min at 10,000 × *g*. The assay mixture had 10% TCA (0.25 mL), phosphate buffer (7.4 pH, 0.2 molar, and 0.2 mL), 0.2 mL phosphoric acid, and 3% of 0.1 mL FeCl_3_. The supernatant was used for absorbance reading at 525 nm on a spectrophotometer. Total AsA was determined by incorporating dithiothreitol (10 mmol L^–1^) into the assay mixture, and dehydroascorbic acid (DHA) was calculated by AsA subtraction from the total AsA. The analyses were carried out in triplicate, and AsA and DHA were reported as mg kg^–1^ FW.

### Superoxide Dismutase, Catalase, and Ascorbate Peroxidase Enzyme Activity Determination in Peel Tissues

Banana peel tissue samples (1 g) were macerated in chilled phosphate buffer (7.2 pH and 100 mmol L^–1^) and centrifuged for 5 min at 10,000 × *g*. The supernatant was used for the determination of CAT and SOD activities. The detailed assay of Ali et al. (2016) was utilized for the determination of enzymes activities of CAT and SOD. The absorbance of CAT enzyme was read at 240 nm. The absorbance of SOD was read at 560 nm. Nakano and Asada (1987)’s protocol was used for APX activity assay, and the absorbance of APX assay was read at 290 nm.

### Glutathione Reductase, Dehydroascorbate Reductase, and Monodehydroascorbate Reductase Enzyme Activity Assays in Peel Tissues

The activity of GR was assessed by adopting the protocol of Parvin et al. (2019). The enzyme reaction mixture consisted of 50 μL crude extract, 50 μmol L^–1^ NADPH, 0.1 mmol L^–1^ EDTA, and 0.5 mmol L^–1^ oxidized glutathione. The absorbance was observed at 340 nm. DHAR enzyme activity was quantified with the assay of Dalton et al. (1986). The assay mixture contained reduced glutathione (2.5 mmol L^–1^), 100 μL enzyme extract, 0.1 mmol L^–1^ EDTA, 0.1 mmol L^–1^ glutathione, and 50 mmol L^–1^ phosphate buffer (7.0 pH). For DHAR, absorbance was noted at 265 nm. MDHAR activity was assayed using the method of Aravind and Prasad (2005). The reaction mixture contained ascorbic acid (7.5 mmol L^–1^), NADPH (2 mmol L^–1^), 25 mmol L^–1^ phosphate buffer (7.0 pH), and 200 μL enzyme extract.

### Determination of Protein Contents in Peel Tissues

The protocol of Bradford (1976) was used for protein content estimation, and the activities of antioxidative and ascorbate glutathione cycle enzymes were expressed as U kg^–1^ protein.

### Statistical Analysis

The work was performed according to a completely randomized design with a factorial layout. The treatments and storage days were considered as factors. The statistical differences among means of the treatments were separated using the least significant difference test (LSD, *P* ≤ 0.05). Statistix^®^ (Version 8.1, Tallahassee, United States) was used for analyzing the collected data.

## Results

### Chilling Injury Index of Banana Fruit

**FIGURE 1:**
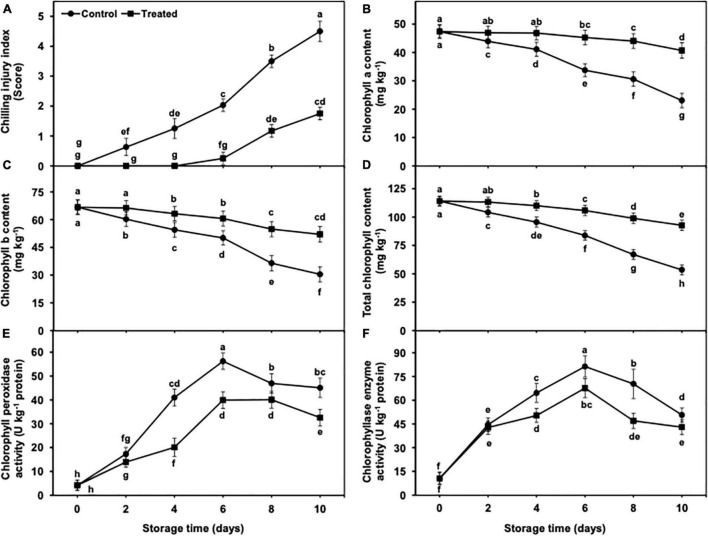
Effect of hydrogen sulfide on chilling injury index **(A)**, chlorophyll a content **(B)**, chlorophyll b content **(C)**, total chlorophyll content **(D)**, chlorophyll peroxidase **(E)** and chlorophyllase **(F)** enzyme activity in peel of banana fruit. Data are mean of three replications and vertical bars show standard of the means. Different letters show significant difference according to least significant difference test.

The incidence of CI represents the severity and symptoms of susceptible produce on its surface. In the current work, CI incidence showed a gradual and significant increase from days 2 to 10 of cold storage (Figure 1A). It is important to mention that no CI was noted in hydrogen sulfide-fumigated bananas till day 4 of cold storage in comparison with control. On day 10, the untreated banana fruits exhibited significantly higher CI incidence (2.57-fold) on the peel surface than the hydrogen sulfide-fumigated group (Figure 1A).

### Chlorophyll Contents in Peel Tissues

The Chl concentrations (Chl b, Chl a, and total Chl) showed a progressive decrease with the advancement of cold storage in both hydrogen sulfide-fumigated and untreated banana fruits (Figures 1B–D). The degradation of Chl contents from days 2 to 10 was markedly less in hydrogen sulfide-fumigated bananas than in untreated fruits. After 10 days, Chl b, Chl a, and total Chl contents were substantially higher (2.04, 1.69, and 1.81-fold, respectively) in the peel of hydrogen sulfide-treated banana fruits than in the untreated group (Figures 1B–D).

### Chlorophyll Peroxidase and Chlorophyllase Activity in Peel Tissues

The activity of chlorophyll peroxidase and chlorophyllase enzymes initially increased from days 2 to 6 and showed gradual but significant reduction, regardless of the treatments (Figures 1E,F). However, hydrogen sulfide-fumigated banana fruits exhibited a substantially less increment in chlorophyll peroxidase and chlorophyllase activity (Figures 1E,F). After 10 days, chlorophyll peroxidase (1.38-fold) and chlorophyllase (1.17-fold) enzyme activities were significantly lower in hydrogen sulfide-fumigated banana fruits than in controls (Figures 1E,F).

### L* Values, Browning Degree, and Soluble Quinone Content in Peel Tissues

**FIGURE 2:**
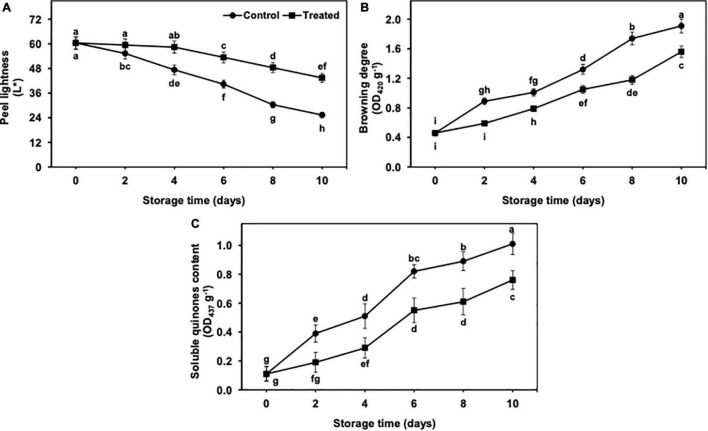
Effect of hydrogen sulfide on peel lightness **(A)**, browning degree **(B)** and soluble quinones content **(C)** in peel of banana fruit. Data are mean of three replications and vertical bars show standard of the means. Different letters show significant difference according to least significant difference test.

The value of L* showed a gradual and significant decrease from days 2 to 10 of the storage, regardless of treatments (Figure 2A). Nevertheless, reduction in L* was found to be lower in hydrogen sulfide-fumigated banana fruits till day 10. On the other side, untreated bananas showed a significantly higher reduction in L* value than the hydrogen sulfide-fumigated banana group. On day 10, hydrogen sulfide-treated banana fruits exhibited a noticeably higher (1.71-fold) L* value than the untreated group (Figure 2A).

The browning degree and soluble quinone content (SQC) showed an upward trend with the extended period of cold storage from days 2 to 10 (Figures 2B,C). However, hydrogen sulfide suppressed the increase in the degree of browning and SQC. On the other side, control banana fruits exhibited a significantly higher browning degree and SQC than the hydrogen sulfide-fumigated group (Figures 2B,C). After 10 days, the browning degree (1.22-fold) and SQC (1.33-fold) were substantially lower in hydrogen sulfide-fumigated banana fruits than untreated control (Figures 2B,C).

### Hydrogen Sulfide and Its Metabolism-Related Enzyme Activity

#### Endogenous Hydrogen Sulfide Content

**FIGURE 3:**
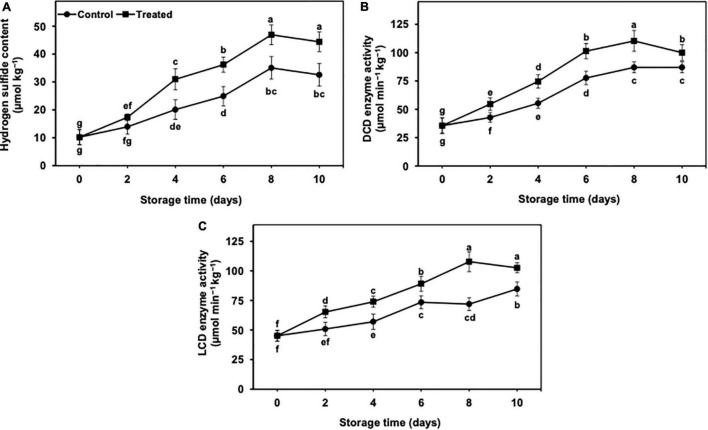
Effect of hydrogen sulfide on endogenous hydrogen sulfide content **(A)**, DCD **(B)** and LCD **(C)** enzymes activity in peel of banana fruit. Data are mean of three replications and vertical bars show standard of the means. Different letters show significant difference according to least significant difference test.

The endogenous hydrogen sulfide content increased with progressed time of storage (Figure 3A). Nevertheless, the increment was significantly higher in treated banana fruits than in untreated control. Overall, hydrogen sulfide increased up to day 8 of the storage in treated banana fruits, whereas in the untreated control, it showed a less increase (Figure 3A). The endogenous hydrogen sulfide decreased in both treated and untreated control bananas on day 10 day of storage, but the decrease was markedly lower in hydrogen sulfide treatment than in the non-treated control. After 10 days, the treated banana fruits showed substantially higher endogenous hydrogen sulfide (1.36-fold) than controls (Figure 3A).

#### Activity of D-Cysteine Desulfhydrase and L-Cysteine Desulfhydrase Enzymes

The activity of DCD and LCD revealed an increasing trend in untreated and hydrogen sulfide-fumigated banana fruits from days 2 to 10 of the cold storage (Figures 3B,C). On an average, untreated control banana fruits exhibited substantially lower DCD and LCD enzyme activity throughout the cold storage (Figures 3B,C). Overall, DCD and LCD enzyme activities remained substantially higher in hydrogen sulfide-fumigated banana fruits till the last day of the cold storage. On day 10, hydrogen sulfide-fumigated bananas revealed significantly higher DCD (1.14-fold) and LCD (1.21-fold) enzyme activity than untreated control (Figures 3B,C).

### Ion Leakage and Malondialdehyde Content

**FIGURE 4:**
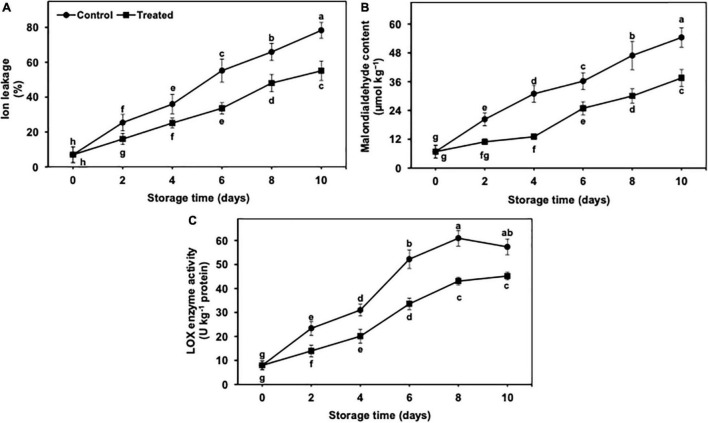
Effect of hydrogen sulfide on ion leakage **(A)**, malondialdehyde content **(B)** and LOX enzyme activity **(C)** in peel of banana fruit. Data are mean of three replications and vertical bars show standard of the means. Different letters show significant difference according to least significant difference test.

The ion leakage and MDA content progressively incremented during 10 days of cold storage in both untreated and hydrogen sulfide-fumigated banana fruits (Figures 4A,B). Nevertheless, hydrogen sulfide-fumigated banana fruits had markedly lower MDA and ion leakage days from 2 to 10 of cold storage in contrast with control. After 10 days, hydrogen sulfide-fumigated banana fruits showed substantially lower ion leakage (1.42-fold) and MDA content (1.44-fold) than the untreated group (Figures 4A,B).

### Lipoxygenases Enzyme Activity

It revealed a significant and gradual increase throughout 10 days of cold storage (Figure 4C). Nevertheless, hydrogen sulfide-fumigated banana fruits had markedly lower LOX enzyme activity than the control. The activity of the LOX enzyme was slightly decreased in the control fruits on day 10, whereas no such decrease was noted in the hydrogen sulfide-fumigated group. After 10 days, hydrogen sulfide-fumigated banana fruits exhibited a substantially lower (1.26-fold) activity of LOX enzyme than control fruits (Figure 4C).

### Hydrogen Peroxide and Superoxide Anion Contents

**FIGURE 5:**
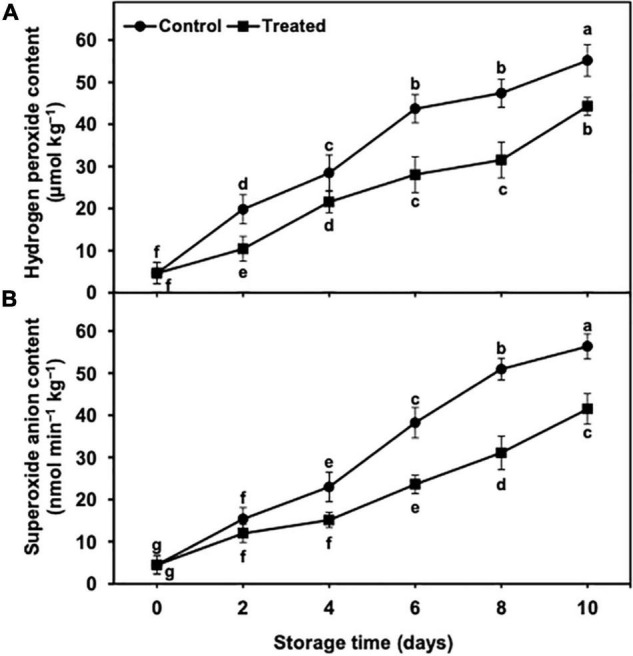
Effect of hydrogen sulfide on hydrogen peroxide content **(A)** and superoxide anion content **(B)** in peel of banana fruit. Data are mean of three replications and vertical bars show standard of the means. Different letters show significant difference according to least significant difference test.

The hydrogen peroxide content showed an increasing trend during the cold storage period of 10 days (Figure 5A). However, hydrogen sulfide application to banana fruits significantly reduced the increase in the hydrogen peroxide content from days 2 to 10 of the cold storage (Figure 5A). On day 10, the hydrogen peroxide content was substantially lower (1.24-fold) in hydrogen sulfide-fumigated bananas than in the untreated group (Figure 5A).

The superoxide anion content indicated an increasing trend from days 2 to 10 of the cold storage time in control and hydrogen sulfide-fumigated banana fruits (Figure 5B). Nevertheless, the superoxide anion content was found to be markedly lower in banana fruits fumigated with hydrogen sulfide than in the untreated group (Figure 5B). After 10 days of cold storage, hydrogen sulfide-fumigated banana fruits revealed a significantly lower (1.39-fold) content of superoxide anion than untreated fruits (Figure 5B).

### γ-Aminobutyric Acid Shunt Pathway in Peel Tissues

#### γ-Aminobutyric Acid Content

**FIGURE 6:**
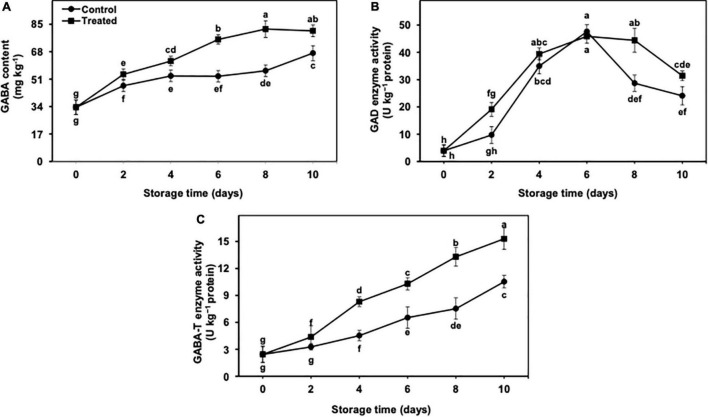
Effect of hydrogen sulfide on GABA content **(A)**, GAD enzyme **(B)** and GABA-T enzyme **(C)** activity in peel of banana fruit. Data are mean of three replications and vertical bars show standard of the means. Different letters show significant difference according to least significant difference test.

The GABA content revealed an increasing trend in untreated and hydrogen sulfide-fumigated banana fruits from day 2 to day 10 of the cold storage (Figure 6A). On an average, the GABA content was continuously increased till day 8 in hydrogen sulfide-fumigated banana fruits and showed a slight reduction on day 10 of cold storage (Figure 6A). Overall, the GABA content remained substantially higher in hydrogen sulfide-fumigated banana fruits till the last day of the cold storage. On day 10, hydrogen sulfide-fumigated bananas revealed a significantly higher (1.21-fold) GABA content than the untreated control (Figure 6A).

#### Glutamate Decarboxylase and GABA Transaminase Enzyme Activity

The accumulation of the GABA content depends upon the activity of GAD and GABA-T enzymes. In the current work, GAD activity was significantly increased in both treatments till day 6 and decreased later from days 8 to 10 (Figure 6B). On day 10, GAD activity was noticeably higher (1.30-fold) in hydrogen sulfide-fumigated banana fruits than in control (Figure 6B). By contrast, GABA-T activity presented a progressive increase in hydrogen sulfide-fumigated and untreated banana fruits throughout the cold storage period of 10 days (Figure 6C). In general, the activity of GABA-T remained markedly higher in hydrogen sulfide-fumigated banana fruits than in the untreated group. After 10 days, hydrogen sulfide-treated fruits exhibited significantly higher GABA-T activity (1.45-fold) than control bananas (Figure 6C).

### Proline Metabolism in Peel Tissues

#### Proline Content

**FIGURE 7:**
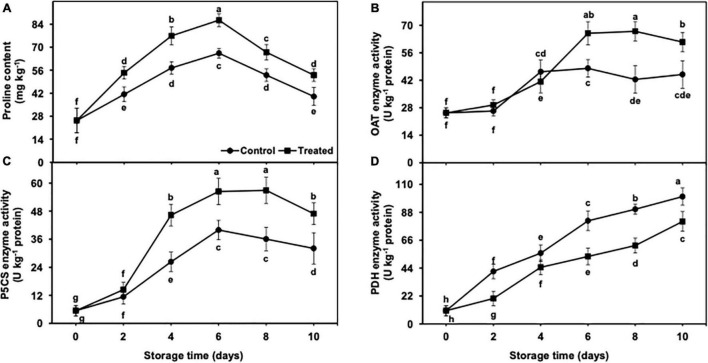
Effect of hydrogen sulfide on proline content **(A)**, OAT **(B)**, P5CS **(C)** and PDH **(D)** enzymes activity in peel of banana fruit. Data are mean of three replications and vertical bars show standard of the means. Different letters show significant difference according to least significant difference test.

The accumulation of the proline content was increased up to day 6 of the cold storage in untreated and hydrogen sulfide-fumigated fruits and presented a rapid decrease on days 8 and 10 (Figure 7A). Nevertheless, the decrease in the proline content was substantially higher in untreated banana fruits. After 10 days, hydrogen sulfide-fumigated banana fruits showed a markedly higher proline content (1.33-fold) than the untreated control (Figure 7A).

#### Δ^1^-Pyrroline-5-Carboxylate Synthetase, Ornithine Aminotransferase, and Proline Dehydrogenase Enzyme Activity

OAT and P5CS activity increased till day 8 in hydrogen sulfide-fumigated banana fruits, and it was decreased on the last day of the storage (Figures 7B,C). By contrast, the untreated group of bananas showed an increase in OAT and P5CS activity up to day 6, and thereafter, a significantly higher decline was noted in the activity of OAT and P5CS enzymes in the control group from day 8 to 10. On an average, hydrogen sulfide-fumigated banana fruits revealed significantly higher OAT (1.36-fold) and P5CS (1.46-fold) activity after 10 days of the cold storage in contrast to the untreated group (Figures 7B,C).

PDH enzyme activity indicated an increasing trend from days 2 to 10 of the cold storage in control and hydrogen sulfide-fumigated banana fruits (Figure 7D). Nevertheless, PDH enzyme activity was found to be markedly lower in fruits fumigated with hydrogen sulfide than in the untreated group. After 10 days, hydrogen sulfide-fumigated banana fruits showed a markedly lower PDH enzyme (1.24-fold) activity than the untreated control (Figure 7D).

### Phenol Metabolism in Peel Tissues

#### Total Phenolic Contents

**FIGURE 8:**
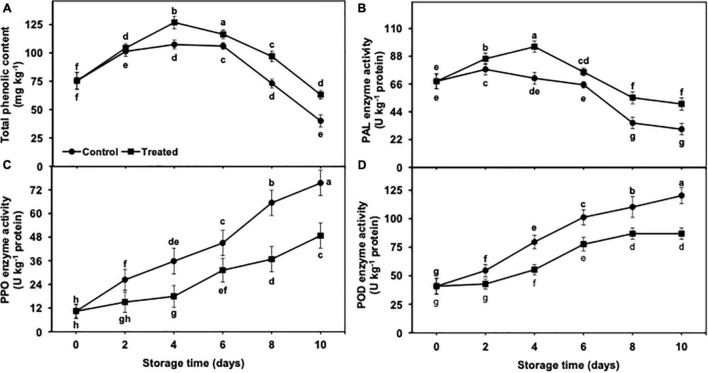
Effect of hydrogen sulfide on total phenolic content **(A)**, PAL **(B)**, PPO **(C)** and POD **(D)** enzymes activity in peel of banana fruit. Data are mean of three replications and vertical bars show standard of the means. Different letters show significant difference according to least significant difference test.

The accumulation of total phenolic content was increased up to day 4 of the cold storage in the untreated group as well as banana fruits fumigated with hydrogen sulfide and presented a progressive decrease from days 6 to 10 (Figure 8A). The decrease in the total phenolic content was markedly lower in hydrogen sulfide-fumigated banana fruits than in the untreated control. In general, hydrogen sulfide-fumigated banana fruits showed a significantly higher (1.57-fold) total phenolic content on day 10 than the control (Figure 8A).

#### Phenylalanine Ammonia Lyase Enzyme Activity

PAL enzyme activity increased till day 4 and then declined from days 6 to 10. It is important to mention that hydrogen sulfide fumigation stimulated the increase in PAL enzyme activity (Figure 8B). On the other side, PAL activity was increased on day 2 and then progressively decreased till day 10 in the untreated control group as compared with hydrogen sulfide treatment. On an average, PAL enzyme activity remained markedly higher in the hydrogen sulfide-treated banana group than in untreated control and exhibited 1.66-fold higher activity of PAL enzyme after 10 days of the storage period (Figure 8B).

#### Peroxidase and Polyphenol Oxidase Enzyme Activity

PPO and POD enzyme activities showed an upward trend with the extended period of cold storage days from 2 to 10 (Figures 8C,D). However, hydrogen sulfide suppressed the increase in POD and PPO enzyme activity. By contrast, the control banana fruits exhibited significantly higher PPO (1.55-fold) and POD (1.38-fold) activity than the hydrogen sulfide-fumigated group (Figures 8C,D). On the other hand, PPO and POD enzyme activity was markedly lower in the hydrogen sulfide-treated group than in untreated bananas after a cold storage period of 10 days (Figures 8C,D).

### Ascorbic Acid, Glutathione, and Dehydroascorbic Acid Contents in Peel Tissues

**FIGURE 9:**
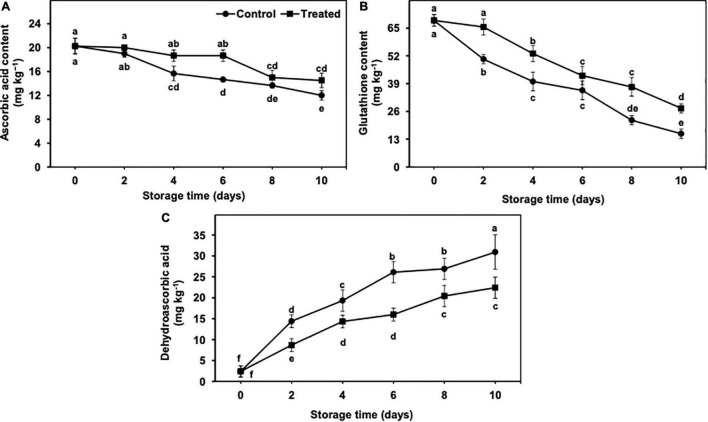
Effect of hydrogen sulfide on ascorbic acid **(A)**, glutathione **(B)** and dehydroascorbic acid **(C)** contents in peel of banana fruit. Data are mean of three replications and vertical bars show standard of the means. Different letters show significant difference according to least significant difference test.

AsA and GSH contents exhibited a gradual but significant reduction from days 2 to 10 of the cold storage, irrespective of the treatments (Figures 9A,B). In general, the reduction in AsA and GSH contents remained markedly less in hydrogen sulfide-fumigated banana fruits than in the untreated control group (Figures 9A,B). It is important to mention that GSH decreased at a significantly higher rate than AsA, regardless of the treatments. On day 10, AsA and GSH were substantially higher (1.21-fold and 1.77-fold, respectively) in hydrogen sulfide-fumigated banana fruits than in the untreated group (Figures 9A,B).

The DHA content showed an increasing trend from days 2 to 10 of the storage period in control and hydrogen sulfide-fumigated banana fruits (Figure 9C). Nevertheless, the increase in the DHA content was markedly lower in banana fruits fumigated with hydrogen sulfide than in the untreated group (Figure 9C). After 10 days of storage, hydrogen sulfide-fumigated banana fruits showed a significantly lower DHA content (1.38-fold) than untreated controls (Figure 9C).

### Superoxide Dismutase, Catalase, and Ascorbate Peroxidase Enzyme Activity in Peel Tissues

**FIGURE 10:**
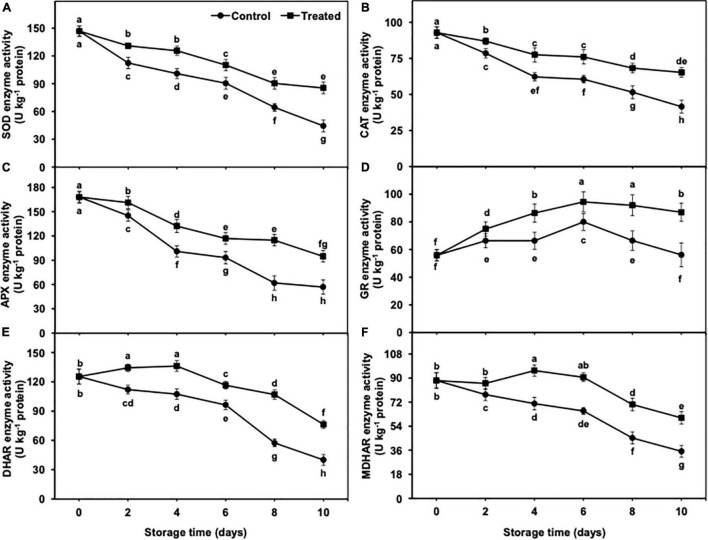
Effect of hydrogen sulfide on SOD **(A)**, CAT **(B)**, APX **(C)**, GR **(D)**, DHAR **(E)** and MDHAR **(F)** enzymes activity in peel of banana fruit. Data are mean of three replications and vertical bars show standard of the means. Different letters show significant difference according to least significant difference test.

The activity of SOD, CAT, and APX showed a gradual and significant decrease from days 2 to 10 of the storage, regardless of treatments (Figures 10A–C). Nevertheless, reduction in SOD, CAT, and APX activity persisted to be lower in hydrogen sulfide-fumigated banana fruits till day 10. On the other side, untreated bananas showed a significant reduction in activity of the aforementioned antioxidative enzymes as compared with the hydrogen sulfide-fumigated banana group. On day 10, hydrogen sulfide-treated banana fruits presented noticeably higher SOD (1.92-fold), CAT (1.57-fold), and APX (1.66-fold) activity than the untreated group (Figures 10A–C).

### Glutathione Reductase, Dehydroascorbate Reductase, and Monodehydroascorbate Reductase Enzyme Activity in Peel Tissues

GR, DHAR, and MDHAR activities showed an initial increase from days 2 to 6 and showed a reduction on days 8 and 10 in all treatments (Figures 10D–F). Nevertheless, the activities of GR, DHAR, and MDHAR remained substantially higher in hydrogen sulfide-fumigated banana fruits than in the untreated control. After 10 days, the hydrogen sulfide-fumigated banana group exhibited substantially higher GR (1.55-fold), DHAR (1.91-fold), and MDHAR (1.71-fold) activity than the control (Figures 10D–F).

## Discussion

Banana is a tropical fruit. The low temperature-induced CI is one of the major problems in the storage and transportation of bananas worldwide. Banana shows CI in the form of browning/surface discolorations of the vascular tissues (Wang et al., 2022). The primary symptoms of CI develop upon the storage of banana fruits under chilling stress after 4 days (Khademi et al., 2019). The increased CI generally leads to discoloration into brown pigments and ultimately results in the loss of peel Chl contents. The reduction in Chl contents occurs during CI stress owing to their degradation (Wang et al., 2016). The degradation of Chl involves chlorophyll catabolism enzymes. It has been reported that chlorophyll peroxidase and chlorophyllase catabolize chlorophyll pigments. In our case, untreated control banana fruits showed significantly higher activity of chlorophyllase and chlorophyll peroxidase enzymes, which thereby resulted in a prompt reduction of Chl pigments (Pongprasert et al., 2011). On the other side, hydrogen sulfide fumigation significantly inhibited the increase in chlorophyll peroxidase and chlorophyllase activities, which in turn conserved better green color of the treated banana peel under postharvest chilling stress.

The appearance and color of banana peel are the major factors that determine the changes in visual quality (Chen et al., 2022). The color of banana peel generally turns brown under chilling stress due to the degradation of Chl contents and the accumulation of brown pigments such as soluble quinones (Pongprasert et al., 2011; Tian et al., 2022). The peel Chl contents degrade probably owing to the increased activity of chlorophyll catabolism-associated enzymes (Pongprasert et al., 2011) and oxidation of phenolics under the action of PPO and POD (Nguyen et al., 2003; Kim et al., 2022). In the current work, hydrogen sulfide-fumigated banana fruits exhibited significantly lower chlorophyll-degrading enzyme activity, soluble quinones, and browning degree. Chilling-induced discoloration of banana peel was significantly lower in hydrogen sulfide-fumigated fruits probably owing to the suppressed PPO and POD activity, which thereby decreased the browning degree, and soluble quinone content possibly due to the protective effect of hydrogen sulfide, which reduced brown pigments formation (soluble quinone content) due to the inhibited increase in the activity of POD and PPO enzymes.

Hydrogen sulfide is an imperative signaling molecule that accumulates during various abiotic stresses in crop plants (Arif et al., 2021). The increased accumulation of hydrogen sulfide ultimately helps mitigate the negative effects of oxidative damage, which occurs under chilling conditions (Aghdam et al., 2018). In order to better understand, we assayed the hydrogen sulfide-biosynthesizing enzymes, that is, DCD and LCD. It was observed that the exogenous treatment significantly stimulated the activity of DCD and LCD enzymes, which ultimately resulted in the increased endogenous hydrogen sulfide concentration. Therefore, it could be considered beneficial and imperative that exogenous application eventually helps increase the endogenous hydrogen sulfide content owing to the enhanced DCD and LCD activity (Li et al., 2016; Liu et al., 2017; Aghdam et al., 2018).

The increased ROS accumulation often leads to indirect or direct oxidative damage to cellular membranes and associated components due to decompartmentalization, and the lipids of the membranes often become leaky (Wang et al., 2016; Ge et al., 2020). Therefore, ion leakage and MDA are considered imperative oxidative damage indicators (Ali et al., 2022). In the present study, hydrogen sulfide-fumigated banana fruits showed markedly lower ion leakage and MDA, which could be owing to the conserved membrane integrity as well as suppressed decompartmentalization, which consequently resulted in a lower increase in MDA production. Hydrogen peroxide (H_2_O_2_) and superoxide anion (O_2_^•⁣–^) are ROS molecules that detrimentally affect the fresh produce owing to oxidative damage (Zhong et al., 2021). The higher H_2_O_2_ and O_2_^•⁣–^ concentrations often lead to membrane disruption and peroxidation of the membrane lipids. In the current work, hydrogen sulfide-treated banana fruits exhibited markedly lower generation of H_2_O_2_ and O_2_^•⁣–^. The lower hydrogen sulfide-induced generation of H_2_O_2_ and O_2_^•⁣–^ may be ascribed to the higher activities of antioxidative enzymes, which probably aided in the efficient scavenging of the overproduced ROS in the treated bananas. Similar suppressed generation of H_2_O_2_ was noted in hydrogen sulfide-treated hawthorn (Aghdam et al., 2018) and persimmon (Niazi et al., 2021) fruits. In the same way, a suppressed O_2_^•⁣–^ content was observed in hydrogen sulfide-treated litchi fruits (Deshi et al., 2020; Siddiqui et al., 2021).

LOX enzyme plays a critical role in the degradation of the lipids in membranes (Xu et al., 2008). The increased LOX activity often leads to higher peroxidative damage to the membrane’s lipid contents and results in the enhanced fluidity of membranes under CI stress (Cao et al., 2009; Wang et al., 2016). In the present investigation, hydrogen sulfide-treated banana fruits exhibited a markedly lower increase in LOX enzyme activity, which could be highly appropriate to conserve membrane integrity and keep the lipid peroxidation under permissible limits, which subsequently helped in the lower expression of CI symptoms on the banana fruits surface.

GABA is an important osmolyte whose changes occur during abiotic and biotic stresses. Its higher accumulation has been found beneficial for the amelioration of CI in various fruits and vegetables. GABA shunt is derived from glutamate for the biosynthesis of endogenous GABA under chilling stress (Wang et al., 2016). In the current work, hydrogen sulfide pretreatment showed a higher accumulation of GABA in banana peel, which appropriately mitigated CI. The metabolism of GABA takes place by the action of GAD and GABA-T enzymes (Ali et al., 2022). In general, GAD and GABA-T enzymes promote GABA accumulation through glutamate (Bhardwaj et al., 2022). The increased GABA accumulation ultimately helps in the alleviation of CI symptoms as noted in tomato (Sharafi et al., 2019), aonla (Ali et al., 2022), and mango (Bhardwaj et al., 2022), where the net accumulation of endogenous GABA was significantly higher in treated produce due to enhanced GAD and GABA-T activity.

Proline is an imperative osmolyte that protects the treated commodity against the chilling-induced negative effects by protecting the membranes from disintegration and lipid peroxidation (Verbruggen and Hermans, 2008). Its endogenous accumulation generally depends on the biosynthesizing and degrading enzymes of proline. The osmotic adjustment due to the accumulation of osmolytes is an imperative defense response of chilling-sensitive produce under cold storage (Ali et al., 2022). In our findings, proline accumulation occurred at a higher rate in hydrogen sulfide-fumigated banana than in control. The accumulation of proline takes place through the OAT and P5CS metabolized pathways (Verbruggen and Hermans, 2008; Kong et al., 2020). On the other hand, PDH degrades proline into glutamate, and it is considered a rate-limiting enzyme (Bhardwaj et al., 2022). In our work, hydrogen sulfide treatment maintained higher P5CS and OAT and lower PDH enzyme activity, which eventually resulted in a higher accumulation of proline in banana fruits during chilling stress.

The phenolic contents are capable of reducing oxidative stress due to ROS scavenging potential (Aghdam et al., 2019). The higher concentration of phenolics is considered beneficial for attenuating the peroxidation of membrane lipids, eventually ensuring CI tolerance (Niazi et al., 2021). PAL enzyme is an important part of the phenylpropanoid pathway, which is considered responsible for the biosynthesis of phenolics. By contrast, PPO is a pro-oxidative enzyme that degrades phenolics by their oxidation combined with POD. The higher PPO and POD activity negatively affect produce visual quality as oxidation of phenols generally results in brownish (owing to soluble quinone content production) coloration (Aghdam et al., 2018; Ali et al., 2019). In the present study, hydrogen sulfide fumigation to banana fruits resulted in substantively lower POD and PPO activity and noticeably higher PAL activity, which therefore led to a higher phenol content, than the control. Therefore, hydrogen sulfide application could be an effective approach for CI mitigation of banana fruits under cold storage.

The fresh produce exposure to chilling generally results in oxidative stress-induced detrimental changes, along with a significant reduction in antioxidant enzyme activity (Xu et al., 2008). The antioxidant enzymes are imperative components of cellular defense against chilling-induced oxidative stress. Among the different enzymatic antioxidants, SOD enzyme dismutases O_2_^•⁣–^ into H_2_O_2_ and H_2_O, whereas APX and CAT ameliorate oxidative damage by converting H_2_O_2_ to H_2_O *via* AsA–glutathione cycle (Noctor and Foyer, 1998; Wang et al., 2016). In addition to antioxidative enzymes, GSH, DHA, and AsA are considered strong ROS scavenging non-enzymatic antioxidants (Hodges et al., 2004). H_2_O_2_ is scavenged by APX enzyme by producing monodehydroascorbate from AsA, and GSH plays a critical part in generating AsA from DHAR (Foyer and Noctor, 2005). Therefore, both GSH and AsA are imperative in the detoxification of overproduced ROS to alleviate CI symptoms. In our study, hydrogen sulfide-treated bananas revealed markedly higher GSH and AsA, along with lower DHA which correlated with substantially lower CI occurrence, suggesting that the aforementioned antioxidants (GSH, DHA, and AsA) played an effective role in banana fruit CI attenuation. In addition to AsA, DHA, and GSH, GR, DHAR and MDHAR collectively work and scavenge H_2_O_2_. In the AsA–GSH cycle, AsA quenches the detrimental H_2_O_2_ directly or acts as an APX enzyme substrate for cyclic H_2_O_2_ reduction (Cao et al., 2021). It is well established that GSH works as an imperative donor of electrons for DHAR and/or MDHAR to regenerate AsA, which is a substrate of APX enzyme. In the present investigation, hydrogen sulfide-fumigated banana fruits displayed significantly higher antioxidant enzyme activity and the AsA–glutathione cycle. So, owing to the higher activity of the antioxidant enzymes and AsA–glutathione cycle, oxidative stress (in terms of H_2_O_2_ and O_2_^•⁣–^ production) was significantly lower, which probably contributed to the alleviation of banana fruit CI symptoms under low-temperature conditions.

## Conclusion

As a result, hydrogen sulfide efficiently alleviated the symptoms of CI in harvested banana fruits. The treatment of bananas with hydrogen sulfide conserved Chl contents and membrane integrity and suppressed the increase in lipid peroxidation, hydrogen peroxide, and superoxide anion concentrations. The treated fruits showed significantly lower Chl-degrading enzyme activity and exhibited a suppressed browning degree and soluble quinone content. Similarly, hydrogen sulfide treatment enhanced proline accumulation owing to higher OAT and P5CS and lower PDH activity. In addition, hydrogen sulfide-fumigated bananas had significantly lower PPO, LOX, and POD and higher PAL activity, which positively contributed to phenol accumulation. Hydrogen sulfide-fumigated banana fruits showed mitigated oxidative stress due to higher antioxidant enzyme activities and the AsA–glutathione cycle. So, hydrogen sulfide (2 mmol L^–1^) fumigation could be used to alleviate CI in cold-stored bananas.

## Data Availability Statement

The original contributions presented in the study are included in the article/supplementary material, further inquiries can be directed to the corresponding authors.

## Author Contributions

SA and AN: conceptualization. SA, AN, SN, and SE: methodology. MM and MS: software. SA, AN, SN, MS, and MM: data analysis. SA, SN, SE, and AN: writing–original draft. HK, JW, AT, and AA: funding acquisition. All authors have contributed in supervision, writing – review and editing, read and agreed to the published version of the manuscript.

## Conflict of Interest

The authors declare that the research was conducted in the absence of any commercial or financial relationships that could be construed as a potential conflict of interest.

## Publisher’s Note

All claims expressed in this article are solely those of the authors and do not necessarily represent those of their affiliated organizations, or those of the publisher, the editors and the reviewers. Any product that may be evaluated in this article, or claim that may be made by its manufacturer, is not guaranteed or endorsed by the publisher.

## Abbreviations

Chl: chlorophyll
CI: chilling injury
D: day
DCD: D-cysteine desulfhydrase
APX: ascorbate peroxidase
AsA: ascorbic acid
ATP: adenosine triphosphate
CAT: catalase
CO: carbon monoxide
DHA: dehydroascorbic acid
DHAR: dehydroascorbate reductase
FW: fresh weight
GABA: γ-aminobutyric acid
GABA-T: GABA transaminase
GAD: glutamate decarboxylase
GR: glutathione reductase
GSH: glutathione
H_2_O_2_: hydrogen peroxide
LCD: L-cysteine desulfhydrase
LOX: lipoxygenases
MDA: malondialdehyde
MDHAR: monodehydroascorbate reductase
NO: nitric oxide
OAT: ornithine aminotransferase
O_2_^ •⁣–^: superoxide anion
P5CS: Δ ^1^-pyrroline-5-carboxylate synthetase
PAL: phenylalanine ammonia lyase
PDH: proline dehydrogenase
POD: peroxidase
PPO: polyphenol oxidase
ROS: reactive oxygen species
SOD: superoxide dismutase
SQC: soluble quinone content
TCA: trichloroacetic acid.
